# Navigating Challenges: Managing Upper Gastrointestinal Bleeding From Cholecystoduodenal Fistula in an Elderly Patient

**DOI:** 10.7759/cureus.66479

**Published:** 2024-08-08

**Authors:** Adam Mylonakis, Maria Sotiropoulou, Lysandros Karydakis, Andreas Koutsoumpas, Andreas Panagakis, Panagiotis Sakarellos, Dimitrios Schizas, Evaggelos Felekouras, Michail Vailas

**Affiliations:** 1 First Department of Surgery, Laikon General Hospital, National and Kapodistrian University of Athens, Athens, GRC; 2 Third Department of Surgery, Evangelismos General Hospital, Athens, GRC; 3 Academic Department of Gastroenterology, Laikon General Hospital, Athens University Medical School, Athens, GRC; 4 First Department of Surgey, Laikon General Hospital, National and Kapodistrian University of Athens, Athens, GRC

**Keywords:** cholecystitis, hematemesis, gastrointestinal hemorrhage, biliary fistula, cholecystoduodenal fistula

## Abstract

Cholecystoduodenal fistula (CDF) is an uncommon condition characterized by an abnormal connection between the gallbladder and the duodenum, often linked to cholelithiasis. It typically presents with nonspecific symptoms such as abdominal pain and jaundice but can occasionally result in severe upper gastrointestinal (GI) bleeding. This report describes the case of a 94-year-old female who presented with hypovolemic shock and multiple episodes of hematemesis. An upper GI endoscopy confirmed a CDF with active hemorrhage. Due to her comorbidities and poor performance status, an endoscopic approach using hemostatic spray was chosen, resulting in a favorable clinical outcome. The development of CDF is typically a result of chronic gallbladder inflammation and cholecystitis, leading to adhesion and erosion into the duodenum. Diagnosis involves imaging and endoscopic techniques, and management varies based on the patient's condition, encompassing surgical, endoscopic, or conservative approaches. This case highlights the necessity of considering CDF in the differential diagnosis of upper GI bleeding, especially in patients with recurrent cholecystitis, and emphasizes the importance of individualized management strategies. It is notable for the use of a minimally invasive endoscopic technique to manage a high-risk patient, highlighting an alternative to surgical intervention.

## Introduction

Cholecystoduodenal fistula (CDF) is an abnormal communication between the gallbladder and the duodenum and is usually present as a complication of cholelithiasis [1]. It often presents with nonspecific symptoms such as epigastric or right upper quadrant abdominal pain and jaundice. Although CDF can lead to severe complications, it is seldom associated with severe and life-threatening gastrointestinal bleeding [2-4]. 

Management of CDF varies based on patient health and symptom severity. Surgical intervention is often required for significant hemorrhage, but less invasive approaches may be preferable for high-risk patients, such as the elderly or those with multiple comorbidities.

This case report details a 94-year-old female who presented with hypovolemic shock and hematemesis due to CDF. Given her advanced age and comorbidities, an endoscopic approach using hemostatic spray was employed, leading to a favorable outcome. This case underscores the importance of considering CDF in the differential diagnosis of upper GI bleeding and highlights the potential of minimally invasive techniques for managing complex cases in high-risk patients.

## Case presentation

A 94-year-old female patient was referred to our department due to multiple episodes of hematemesis over the last 5 hours. Her medical history included hypertension, atrial fibrillation, and hyperuricemia, for which she was treated with angiotensin-converting enzyme inhibitors, beta-blockers, warfarin, and allopurinol. The patient had no history of cholelithiasis, cholecystitis, or any similar biliary pathology. She also had no previous episodes of gastrointestinal bleeding.

On admission, the patient was lethargic, hypotensive (blood pressure 90/70mmHg), and tachycardic (heart rate 110/min). Her abdominal examination revealed diffuse abdominal guarding and the digital rectal examination was positive for melena. Laboratory investigation showed hematocrit: 24%; white blood cells: 9,700/mm3; platelets: 133,000/mm3; blood urea nitrogen: 68 mg/dl; creatinine: 1.03 mg/dl. The liver function test noted the following: Aspartate aminotransferase: 46 IU/l; Alanine aminotransferase: 69 IU/l; total bilirubin: 0.67 mg/dl; direct bilirubin: 0.34 mg/dl; alkaline phosphatase: 241 IU/l; and γ-GT: 126 U/l. Coagulation parameters were normal, with an International Normalized Ratio (INR) of 1.03 and a Prothrombin Time (PT) of 12.4 s. Initial management included resuscitation with blood products and an intravenous proton pump inhibitor (PPI). Following the initial resuscitation, the patient showed notable improvement, with stabilization of vital signs and improved consciousness.

**Figure 1:**
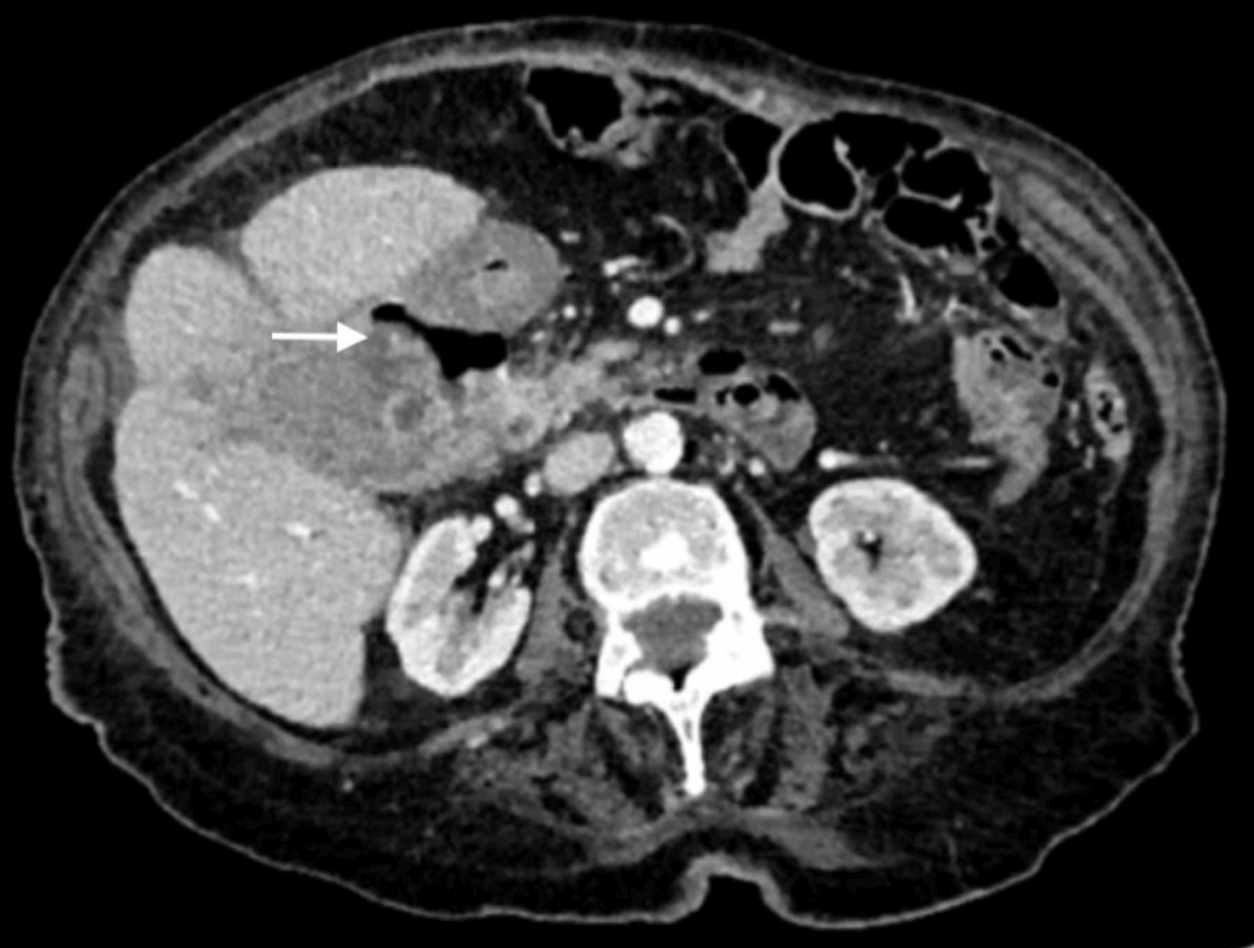
Axial abdominal computed tomography with intravenous contrast shows gallbladder wall thickening with edema. The white arrow indicates the fistulous communication between the gallbladder and the duodenum

The patient underwent an abdominal computed tomography, revealing thickening of the gallbladder, active hemorrhage, and a fistula between the gallbladder and the first part of the duodenum, with no evidence of gallstones (Figure 1). The decision to perform a CT scan before an upper GI endoscopy was based on the need to rapidly identify the source of bleeding and assess the extent of the pathology, given the patient’s presentation with hypovolemic shock and multiple episodes of hematemesis, as well as the greater availability of a CT scan compared to an upper GI endoscopy in our department, facilitating quicker diagnostic assessment in emergencies. The patient’s anticoagulant medication was stopped, and she was given intravenous antibiotics as per local protocol, specifically piperacillin, tazobactam, vancomycin, and fluconazole. 

**Figure 2:**
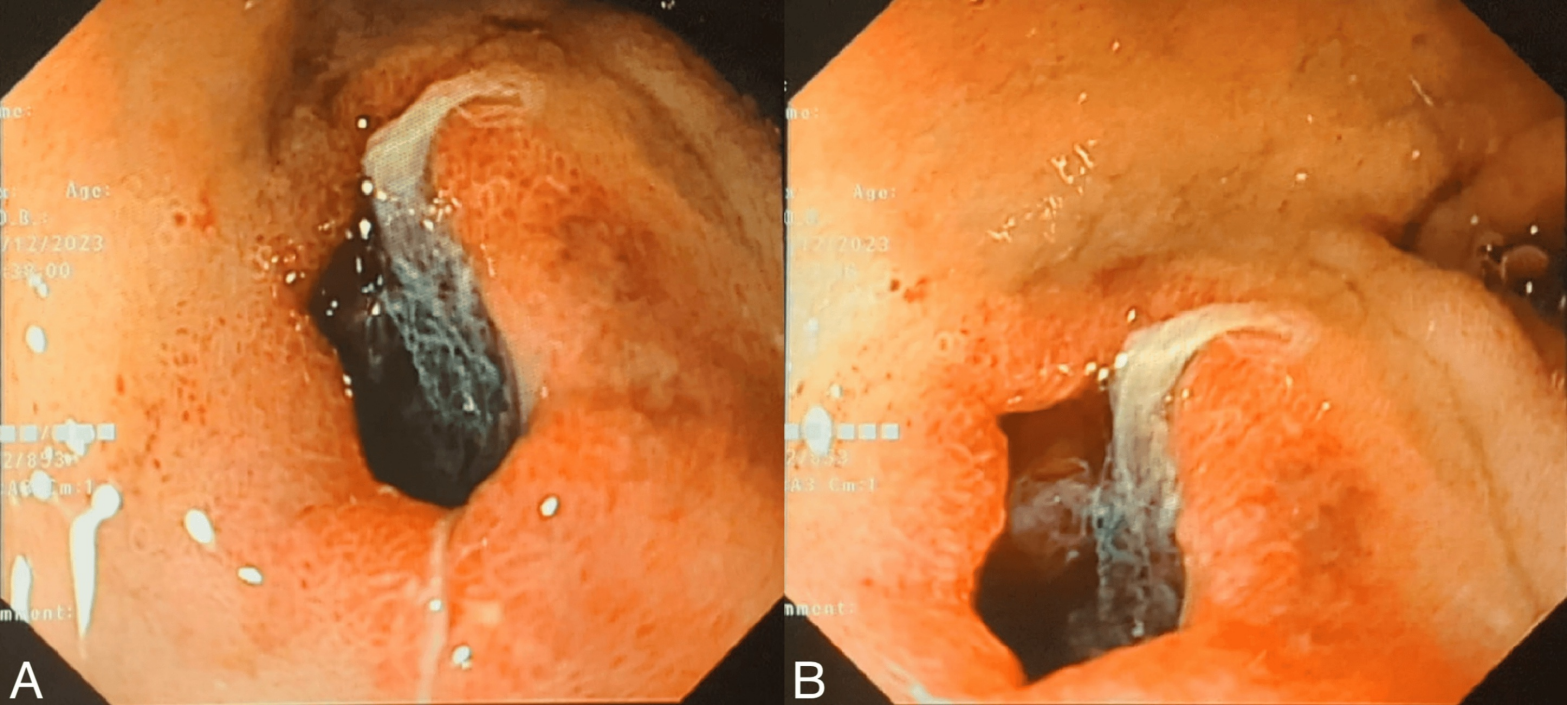
Panels A, B: Endoscopic images showing the duodenal opening of the cholecystoduodenal fistula at the anterior wall of the duodenal bulb, with blood clots in the orifice of the fistula, suggestive of bleeding from the cholecystoduodenal fistula

Due to the patient's high ASA (American Society of Anesthesiologists) score of IV and a Charlson Comorbidity Index (CCI) of seven, indicating significant comorbidities and poor performance status, an endoscopic approach was preferred over surgical intervention. The patient underwent an esophagogastroduodenoscopy, which revealed an orifice (measuring 8 mm) at the anterior wall of the duodenal bulb with blood clots suggestive of bleeding from a cholecystoduodenal fistula (Figure 2). After rinsing the blood clots, hemostat powder spray was applied. The use of endoclips or over-the-scope clips was deemed risky as the orifice was 8 mm in diameter, and there was no evidence of active bleeding at the time of the procedure. After this intervention, the patient was stabilized, and melena stopped with no evidence of GI bleeding.

The patient's post-intervention was complicated by a pleural effusion, which was drained under ultrasound guidance. She was discharged 10 days after admission with oral antibiotics and PPIs. During her last follow-up three months later, the patient was doing well with no abdominal symptoms or bleeding signs.

## Discussion

Cholecystoduodenal fistula (CDF) is an exceedingly rare clinical entity characterized by abnormal communication between the gallbladder and the duodenum. It accounts for approximately 80% of cholecystoenteric fistulas, a condition with reported incidences ranging from 0.15% to 5% of biliary disease [5]. The development of CDF is predominantly associated with gallstone disease, which accounts for more than 90% of spontaneous instances. The remainder of cases are secondary to peptic ulcer disease or malignancies involving the stomach, gallbladder, pancreas, duodenum, or colon [1,2]. A minority of CDF instances may be iatrogenic, consequent to the deployment of self-expandable metallic biliary stents (SEMS), used for treating acute cholecystitis in patients who are not suitable for surgical intervention [3]. In the case presented, the patient exhibited no history of cholelithiasis, and computed tomography scanning failed to reveal any gallstones, despite a high index of clinical suspicion for gallstone cholecystitis.

The pathogenesis of CDF is intricately linked to chronic inflammatory processes of the gallbladder, mainly caused by recurrent episodes of cholecystitis. This chronic inflammation predisposes to adhesion formation in the duodenum, leading to eventual erosion and fistula development. Clinical manifestations of CDF are nonspecific, often simulating chronic biliary pathology, including pain in the right hypochondrium, epigastric discomfort, recurrent cholangitis, pancreatitis, and gallstone ileus. Although fistulae can theoretically predispose to ascending infections within the biliary tract, such occurrences are reported infrequently, rendering high fever an atypical manifestation [4]. Hemorrhage, potentially resulting from erosion into the cystic artery, presents even more rarely. To our knowledge, this is the 14th case of spontaneous cholecystoduodenal fistula presenting as upper GI hemorrhage reported in the literature [6-15]. The development of the CDF in this case was likely due to acalculous cholecystitis, which involves inflammation of the gallbladder without the presence of gallstones.

Diagnosing CDF, particularly when presenting emergently as hemorrhage, relies on a multifaceted approach incorporating several diagnostic modalities. Endoscopic retrograde cholangiopancreatography (ERCP) and upper GI endoscopy, in particular, provide the benefit of directly visualizing the leakage of contrast media from the gallbladder into the duodenum, offering conclusive evidence of fistula presence. However, the effectiveness of ERCP depends on the patency of the cystic duct, with the procedure bearing a potential risk for ascending infections post-manipulation [16]. Computed tomography characteristically reveals morphological aberrations of the gallbladder and bile ducts, such as gallstones, gallbladder edema or atrophy, intraluminal gas, ectopic intestinal stones, mechanical intestinal obstruction, and biliary pneumatosis. Notably, clinicians must distinguish these findings from those of Mirizzi syndrome, which is marked by biliary dilatation without evidence of gas accumulation or a gallbladder-intestine fistula [17].

The ultrasonic features of CDF include a variety of distinctive findings. Atrophic changes in the gallbladder manifest as thickening of the wall and disappearance of the normal anechoic zone, indicating altered morphology. Intra- and extra-hepatic biliary pneumatosis is characterized by cord-like, strong echoes within the biliary duct. Adhesions between the gallbladder and duodenum, where the adherent tubal wall appears thinner, exhibit low echoes and partial defects. Upon deep breathing, synchronized movement is observed between the gallbladder and the superior liver, alongside the inferior duodenum, demonstrating the same range of motion. Dynamic changes in the morphology and volume of the gallbladder and the adherent duodenum occur following the administration of oral contrast, which enters the gallbladder through the fistula orifice, with a portion of the contrast flowing into the extrahepatic duct [18]. As far as elective cases of CDF, MRI is increasingly recognized as a critical tool in the preoperative assessment of cholecystoduodenal fistulas, significantly reducing the frequency of intraoperative discoveries by accurately depicting disease extension and aiding in therapy decisions [19].

The primary treatment for symptomatic CDF presenting with upper GI hemorrhage is surgical intervention, as significant bleeding from the cystic artery is unlikely to be managed conservatively or with endoscopic hemostasis [6]. For surgical repair, laparoscopic techniques have been proven to be safe and efficient, with no increase in morbidity risk when performed by a skilled laparoscopic surgeon [20,21]. Various surgical approaches, including cholecystectomy-first and fistula-first repair, have been advocated based on the surgeon's discretion and patient-specific factors [22].

In cases where patients are poor surgical candidates, the use of endoscopic retrograde cholangiopancreatography (ERCP) to perform a biliary sphincterotomy has been suggested. This procedure allows for greater drainage of bile along the body’s natural biliary passages, thus controlling biliary leakage through the fistulous communication between the gallbladder and duodenum. This approach may ultimately facilitate the natural healing of the cholecystoduodenal fistula [23].

Other non-surgical approaches, such as selective embolization of the cystic artery and, in rarer cases, the inferior pancreaticoduodenal artery by interventional radiologists, have been reported to be successful [7]. Lastly, there are reported cases of patients deemed unfit for any intervention that has had favorable outcomes after spontaneous closure of cholecystoduodenal fistulae [24].

The overall prognosis for patients presenting with upper GI bleeding due to CDF is generally favorable, significantly influenced by the timely and appropriate selection of therapeutic strategies, the patient's underlying health status, and the presence of comorbid conditions. However, there has been one reported case of death due to upper GI bleeding, underscoring the importance of careful monitoring and management in patients with this rare condition [11]. In our specific case, due to the patient's comorbidities and poor performance status, conservative treatment was opted for, utilizing hemostatic spray during upper GI endoscopy, which resulted in a favorable patient outcome and follow-up.

## Conclusions

Cholecystoduodenal fistula, though rare, should be considered a cause of upper GI bleeding in patients presenting with upper abdominal pain and recurrent episodes of cholecystitis. An individualized treatment approach, tailored to patient characteristics and surgeon experience and incorporating the expertise of gastroenterologists and interventional radiologists, is critical for optimal management and patient outcomes.
